# Flow States and Associated Changes in Spatial and Temporal Processing

**DOI:** 10.3389/fpsyg.2020.00381

**Published:** 2020-03-12

**Authors:** Scott Sinnett, Joshua Jäger, Sarah Morgana Singer, Roberta Antonini Philippe

**Affiliations:** ^1^Department of Psychology, University of Hawaii at Manoa, Honolulu, HI, United States; ^2^Institute of Psychology, University of Bern, Bern, Switzerland; ^3^Laboratoire PHASE, Institut des Sciences du Sport, Faculté des Sciences Sociales et Politiques, Université de Lausanne, Lausanne, Switzerland

**Keywords:** flow, temporal processing, spatial attention, hierarchical linear modeling, perception, sport, music, high performance

## Abstract

Improved perception during high performance is a commonly reported phenomenon. However, it is difficult to determine whether these reported changes experienced during flow states reflect veridical changes in perceptual processing, or if instead are related to some form of memory or response bias. Flow is a state in which an individual experiences high focus and involvement in a specific task, and typically experiences a lack of distractibility, a disordered sense of time, great enjoyment, and increased levels of performance. The present pre-registered study investigated 27 athletes and musicians using a temporal order judgement (TOJ) task before and after a sports or music performance over three sessions. Participants' flow experiences were surveyed in order to measure how modulations of flow over successive performances potentially modulates spatiotemporal perception and processing. Hierarchical linear modeling showed a positive moderation of subjectively experienced flow and performance on post-measures of a TOJ task. Specifically, the higher the subjective flow experience of the sport or music performance was rated, the better the participant performed in the post-performance TOJ task compared to the pre-performance TOJ task. The findings of the present study provide a more comprehensive explanation of human perception during flow at high level performances and suggest important insights regarding the possibility of modulated temporal processing and spatial attention.

## 1. Introduction

Several anecdotal claims regarding improved perception during flow states have been reported in various populations, yet it remains an open question as to whether there is a veridical change in perception, or if instead these reported improvements in perception are related to post-performance memory biases. As an example of this supposed improvement in performance, George Scott, a professional baseball player, stated in an interview: “When you're hitting the ball [well], it comes at you looking like a grapefruit. When you're not, it looks like a blackeyed pea” (Witt and Proffitt, 2005, p. 937). In an attempt to disentangle the question of whether perception is indeed modulated during optimum performance levels, Witt and Proffitt (2005) correlated performance when playing softball (i.e., batting averages) and the perceived size of a softball. According to Witt and Proffitt (2005), successful players perceived the ball to be bigger than less successful players, with this finding leading the authors to further claim that enhanced performance levels are indeed capable of modulating perception. Similar findings have been found with darts players, with throwing ability in darts influencing the perceived size of a target (Wesp et al., 2004; Cañal-Bruland et al., 2010), such that participants with better accuracy chose bigger circles corresponding to the size of the target than participants with lower accuracy. In addition, people who perform better in archery report seeing larger targets when compared to their weaker counterparts (Lee et al., 2012), and high-performing golfers (compared to weaker golfers) perceive the size of the cup to be larger (Witt et al., 2008). Moreover, distances are perceived as longer by people who are overweight (Sugovic et al., 2016), by people carrying a heavy backpack (Proffitt et al., 2003), or when throwing a heavy object toward a certain destination (Witt et al., 2004). Witt (2019) provides a review on action-specific effects on modulated spatial perception for a review on action-specific effects on modulated spatial perception.

These types of performance-dependent modulations of perception extend to the temporal domain. For instance, Gray (2013) found that not only was perceived ball size larger amongst high performing baseball players, but also the perceived speed of the ball, which was rated to be slower by better players. Additionally, tennis players who played better than other players perceived the ball to move more slowly (Witt and Sugovic, 2010). This evidence dovetails with the phenomenological experience of time slowing during threatening events (Arstila, 2012) as well as how the prospect of reward can affect the subjective perception of time (Failing and Theeuwes, 2016). Different approaches might offer an explanation for these phenomenological circumstances, of how or why our subjective perception of time changes when performing well or when placed in fear provoking circumstances. One possible explanation could derive from memory biases. For example, it is possible that players who perform poorly could perceive the size of the target during the game as exactly the same size as better players, but recall the size of the targets to be smaller, perhaps as a means to justify their poor performance (Cooper et al., 2012). A memory bias could also lead to the perception that time seems to slow down, because richer than usual memories could later be improperly connected in such a way that they span a longer period than the experience on which they were actually based on (Arstila, 2012). Furthermore, neurophysiological correlates have been identified that are related to perceptual alterations (van der Kruijs et al., 2014) might play a crucial role in experiencing such phenomenological modulations. Ursano et al. (2007) discuss in their investigation about dissociative reactions during traumatic events the contribution of the cerebellum in perceptual alteration concerning time and space.

When conceptualizing the state of the literature that has explored the phenomenological experience of modulated perception (see for example Witt and Proffitt, 2005), it is important to note that mostly intra-individual differences in perception that may result from different levels of performance are not taken into account; instead, most investigations have simply focused on how better players compare to weaker players (e.g., players with better batting averages vs. worse batting averages). It is entirely possible that a better player might perceive the ball or a target to be larger than a weaker player. This essentially equates to a measure of good vs. bad players, and is therefore uninformative with respect to the question of whether or not performing at a high level modulates perception. Furthermore, to the best of our knowledge most researchers have also failed to consider the subjective evaluation that an athlete might have regarding their own personal performance, which could be different from the objective evaluation of, for instance, averaging their hitting rate. With specific respect to Witt and Proffitt (2005), batting average was based on self-report and calculated on a relatively small number of attempts (1–2 games only, without the exact number of at-bat attempts reported). As can clearly be seen, a longitudinal approach to investigating such phenomena is needed.

In addition to investigating potential perceptual modulations that arise due to high performance, phenomenological experiences associated with flow experiences have also been robustly explored. The experience of flow (Jackson and Csikszentmihalyi, 1999; Engeser and Rheinberg, 2008) refers to high performance in a task (e.g., athletics, music, etc.) that often involves increased levels of focus until complete immersion occurs, attention that is not distracted by anything irrelevant, feelings of optimal challenges, and deep enjoyment (Csikszentmihalyi, 1975, 2000). Recent research suggests that flow can be characterized by nine different dimensions (Csikszentmihalyi, 2000, 2002): challenge-skill balance (demanding situations in which the individual is engaged but not overwhelmed to meet the challenge), clear goals that derive from the activity, unambiguous feedback that helps individuals to constantly adapt in order to achieve their goals, concentration on the task at hand (one's focus relies on the activity and is not distracted by irrelevant stimuli), action-awareness merging (total immersion in the activity), loss of self-consciousness (individual's self-awareness and concerns regarding external evaluations decreases), increased sense of control (knowledge about the ability to keep things under control, if necessary), and transformation of time (disordered perception of time). The first three (i.e., challenge-skill balance, clear goals, and unambiguous feedback) are required conditions for flow to occur, while the remaining items refer to the phenomenological characteristics frequently associated with flow. These dimensions have been studied in many different populations mainly using self-report approaches (Moneta, 2012; Swann et al., 2012; Chirico et al., 2015; Stamatelopoulou et al., 2018; Habe et al., 2019).

Of direct concern to the research conducted here, the notion that the perception of time can be modulated when in flow states has been frequently reported, although these reports are almost exclusively anecdotal. For instance, an elite track and field athlete claimed that “When I went to throw it [the javelin], it was like things were in slow motion, and I could feel the position I was in, and I held my position for a long time” (Jackson, 1995, p.82). This statement, and others regarding the altered perception of time, refers to the speed at which the passage of time is experienced (Thönes and Stocker, 2019).

A challenge for the claim that the perception of time slows down during flow states can be found in the difficultly of disentangling the subjectively perceived experience of time from objective perception. It is unlikely that flow states would lead to (or arise from) a speed up in neuronal communication, with the question being further muddled by the fact that attempts to measure perception during flow states would almost surely take the individual out of that state. As such, questions related to time perception during flow states are limited to posteriori surveys, therefore, the underlying processes of any modulation in the perception of the passage of time still remain unknown (Wearden, 2015; Tanaka and Yotsumoto, 2017).

The aim of the present study is to investigate whether potential changes in temporal and spatial processing are modulated by increases in flow experiences. Possible modulations of temporal processing and spatial attention can be measured with a temporal order judgement (TOJ) paradigm, a task that has been widely used as a tool to measure temporal and spatial processing. By means of the TOJ task, two different values can be calculated: The just noticeable difference (JND) and the point of subjective simultaneity (PSS) (West et al., 2008; Lim and Sinnett, 2011). The former is a measure of temporal processing and refers to the smallest amount of time needed to accurately separate two stimuli 75% of the time, and thus be able to correctly identify the order of presentation. The latter is a measure of spatial attention and reflects the extent to which attention is distracted by a spatial cue, either peripheral (exogenous) or central (endogenous), such that the uncued side must be presented before the cued side in order for both stimuli to be perceived as having been presented simultaneously.

The cues in the TOJ task create a prior entry effect (Shore et al., 2001): Attended stimuli are perceived before unattended stimuli, showing that temporal processing is influenced by attention (Shore et al., 2001). By presenting such cues in the TOJ task prior to the onset of the first stimulus, attention should be, at least in theory, directed toward the cued side, resulting in the cued side being detected first, even when both items had been presented simultaneously. That is, if the left and right stimuli appear simultaneously, for example, the stimulus at the cued side will be perceived as having occurred first and the PSS would indicate a shift of attention toward the cued side (Shore et al., 2001). The shift might be greater for exogenous cues than for endogenous cues, due to increased volitional control over orienting effects for central cues (Shore et al., 2001). Notably, evidence has been observed that faster stimulus perception associated with the prior cue reliably results from the allocation of spatial attention and not from any potential response bias (Ulrich, 1987; Stelmach and Herdman, 1991; Shore et al., 2001; West et al., 2009).

The TOJ paradigm has been used in several situations as a viable approach for measuring perception. For example, Lim and Sinnett (2011) showed lower JND scores for musicians, suggesting better temporal discrimination in musicians than in controls. Similarly, modulations in visual attention were observed after extensive action video game play, with West et al. (2008) showing greater sensitivity to exogenous sensory stimuli and the potential that video game play modulates spatial attention. While neither of these studies considered whether temporal perception might be modulated when these groups of participants were in flow states, these modulations in information processing nonetheless are attributable to long-term experience, and on a neurological level, arguably due to increased neuro-plasticity. For instance, Granek et al. (2010) and Gong et al. (2016) used fMRI to show greater activity in the prefrontal cortex within video-game experts during complex non-gaming tasks, and increased functional integration between two critical neural networks for visual attention, namely the salience network and the central executive network, when compared to novices. These different brain patterns can be potentially explained by the task demands on visual attention that are associated with video games. Using behavioral and electrophysiological measures, Qiu et al. (2018) investigated the effects of short- and long-term action video gaming on measures of visual attention. After a short session of playing an action video game, experts and novices showed performance improvements in visual attention, with experts outperforming novices before the session. Importantly, modulated electrophysiological measures in novices were found. These findings provide evidence for a correlation between plasticity of visual attention and action video gaming, even after a brief session of gaming.

In this study we explored whether such modulation of temporal processing and spatial attention can also be observed depending on flow experience, in the short-term. Given the continuum of performance levels within any performer, flow experiences provide an ideal place to investigate how phenomenological experience might possibly modulate perception. While this is clearly the case considering anecdotal evidence, it is unknown whether there are objective enhancements in perception when participants experience a higher feeling of flow compared with when they experience a lower state of flow. By measuring flow levels and temporal processing across multiple sport and music performances in practice or rehearsal sessions, we are able to address this question to an extent that has not been done previously, to the best of our knowledge. Precisely, if individuals in a flow state do experience a slow down in the perception of time, this should be correlated with improved temporal processing of stimuli (i.e., a smaller JND) when in a flow state compared to when they are not performing at that optimal level. Additionally, we extended this question to determine whether individuals in a flow state are less distracted by exogenous or endogenous cues, potentially suggesting enhanced spatial attention when performing in a higher flow state. The present study will help provide a more comprehensive explanation of modulated temporal processing and spatial attention during flow states with intra-individual differences in consideration.

## 2. Materials and Methods

### 2.1. Participants

Eleven athletes (mean age = 23.6, *SD* = 3.53, 4 female and 7 male) and 16 musicians (mean age = 20.8, *SD* = 3, 10 female and 6 male) from various sports and musical disciplines were recruited. One additional subject (athlete) was used for piloting and excluded from the analyses due to irregularities in the testing procedure and refinement of the experiment (e.g., increasing the number of repetitions of the TOJ task). The athletes had 14 (*SD* = 5.87) years of experience and practiced 18 (*SD* = 6.83) hours per week on average. The musicians had 8.8 (*SD* = 2.94) years of experience and practiced 8.5 (*SD* = 6.33) hours per week on average. Due to previous findings suggesting that skill level is correlated with the experience of flow (Catley and Duda, 1997; Engeser and Rheinberg, 2008), expertise was operationally defined as regular practice over several years in a particular discipline. Additionally, all athletes and musicians currently compete or perform at exceptional levels (e.g., NCAA Division II tennis players; performing musicians, etc.). The years of experience between athletes and musicians were significantly different, *t*_(13)_ = 2.72, *p* = 0.017, as well as the weekly practice hours, *t*_(20)_ = 3.65, *p* = 0.002. Altogether, 27 trained musicians and athletes (mean age = 21.9, *SD* = 3.47, 14 female and 13 male) participated. To cover a more general picture about flow across expertise types, a diverse sample of athletes (2 runners, 9 tennis players) and musician types (1 piano, 2 trumpets, 4 flutes, 2 clarinets, 2 saxophones, 1 bassoon, 1 oboe, 1 trombone, 1 tuba, 1 percussion) participated. The study was approved by the University of Hawaii at Manoa's committee on human subjects (CHS). All participants provided written informed consent before beginning the study. In order to compensate for their time, participants were offered the opportunity to participate in a mental preparation seminar held by one of the authors (RAP). Due to drop outs, altogether five experimental runs of two participants are missing.

### 2.2. Task

**Figure 1:**
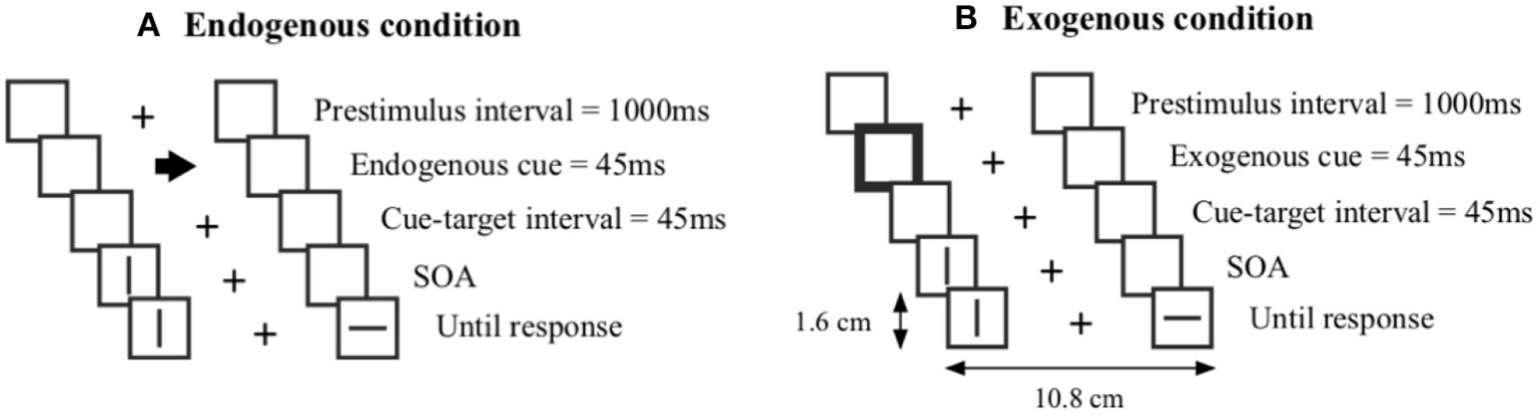
**(A)** Endogenous cues are indicated by an arrow in the middle and **(B)** exogenous cues are indicated by a thick frame. Each trial started with a fixation cross followed by a cue, a cue-target interval followed by the appearance of the first target and a specific stimulus onset asynchrony (SOA) interval followed by the second target.

The experimental task consisted of a temporal order judgment task (TOJ), adapted from Lim and Sinnett (2011), designed to measure temporal processing and spatial attention. Two versions with different conditions of the TOJ task were presented in separate blocks, one with exogenous cues and the other with endogenous cues (Figure 1). The TOJ tasks were presented successively in separate sessions and counterbalanced blocks. Approximately half of the participants started with the endogenous condition. Each trial started with a fixation cross in the middle of the screen flanked by two placeholder squares. The length between the outer ends of the placeholder squares to the fixation cross was 5.4 cm. After 1,000 ms, either an exogenous or an endogenous cue was displayed for 45 ms. Exogenous cues were created by thickening placeholder squares to 4 pixels, whereas endogenous cues consisted of a central arrow (1.2 cm). Following the appearance of the cue with a delay of 45 ms, the first target (horizontal or vertical line) was displayed in either the left or right placeholder square. The other target appeared in the other placeholder square after a specified stimulus onset asynchrony (SOA). Target orientation and appearance side were presented with the same probability of occurrence. The targets (1.2 cm) appeared within the placeholder squares (1.6 × 1.6 cm). Participants were then forced to make a choice on the keyboard to indicate on which side they perceived the target to appear first. To determine the SOAs for each trial, a 1-up-3-down adaptive staircase approach (Cornsweet, 1962) was used. Each block started with a SOA of 267 ms. Depending on whether 3 correct or one incorrect response was given, the SOA would respectively decrease or increase by 16.7 ms on the subsequent trial. Each block was finished after 14 reversals. Altogether, the TOJ task lasted ~7–10 min. The experiment was programmed and run using the software PsychoPy 3.0 (Peirce et al., 2019).

### 2.3. Questionnaire

To measure flow states, the Activity Flow State Scale (AFSS) (Payne et al., 2011a) was used. The AFSS captures the 9 dimensions of flow [Merging actions and awareness (MAA); Clear goals (CG); Concentration on task at hand (CO); Unambiguous feedback (UF); Challenge skill balance (CS); Transformation of time (TT); Sense of control (CN); Loss of self-consciousness (SC); Autotelic experience (AE)] according to Csikszentmihalyi (2000, 2002) with 26 statements. It has a high reliability with Cronbach's alpha coefficients for the 9 subscales ranging from 0.71 to 0.90 (Payne et al., 2011a). It has been shown to measure flow in different populations and was specifically constructed to measure flow across a wide range of activities (Payne et al., 2011b; Osin et al., 2016). The items are rated on a 5-point Likert scale, ranging from 1 (strongly disagree) to 5 (strongly agree). A global flow score (the mean of all items) was computed for each participant.

### 2.4. Design

**Figure 2:**
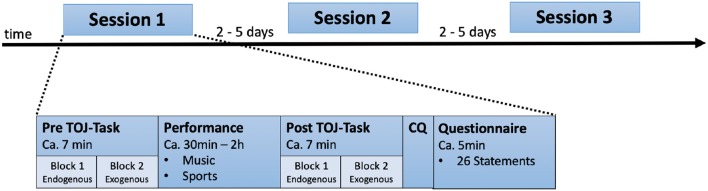
The within-subjects design was divided into three repeated sessions with 2–5 days intervals. One session run is shown as an example: All participants completed a pre-performance TOJ task (including endogenous and exogenous trials), a sports or music practice or rehearsal session, a post-performance TOJ task (including endogenous and exogenous blocks, which were presented in a counterbalanced order between the participants and sessions), a control question and a self-report questionnaire (AFSS, displayed in randomized order).

The experiment was divided into three sessions conducted ~2–5 days apart (Figure 2). In each session, all participants completed a pre-TOJ task, including both endogenous and exogenous conditions, a sports or music performance (practice or rehearsal sessions), a post-TOJ task, again including endogenous and exogenous conditions, a control question and the questionnaire (AFSS) at the end of each session.

### 2.5. Procedure

Participants were recruited according to their level of expertise. All sessions took place next to the participants' practice environment to ensure high ecological validity, and were managed by at least two researchers at a time. In the first session, participants were asked to provide informed consent, and then filled out a questionnaire collecting demographic information, including information about years of experience in their specific discipline and weekly practice amounts. The participants were then seated ~ 60 cm from the monitor. The investigation was set up in a way that a maximum of six people could participate at the same time. Each session started with a written introduction for the upcoming task and a reminder to complete the task as quickly and accurately as possible. As soon as the participants were familiar with the task and felt ready, they began with the pre TOJ task by pressing the space bar on the keyboard. The task was divided into 2 blocks with a short break in between. Each block included either the exogenous or the endogenous condition, each lasting ~ 3–5 min. After the TOJ task, the participants completed their sports or music performance (practice or rehearsal sessions), which lasted between 30 min to 2 h. The duration of the practice or rehearsal sessions depended on the usual practice schedule of the participants. Immediately after the practice or rehearsal session, the participants completed the post-TOJ task (same procedure as the pre-TOJ task) followed by the self-report questionnaire (AFSS) about their flow state during the sports or music session, altogether lasting around 10 min. Prior to beginning the AFSS, a control question ranging from 1 (strongly disagree) to 5 (strongly agree) was inserted in order to get an impression if participant's state, emotions, and body feelings were about the same during the practice or rehearsal session and the post-TOJ task, and intended to gauge whether the participants still experienced flow while doing the post-TOJ task. The task and the questionnaire were presented on 6 different 13.3” laptop computers each with a refresh rate of 60 Hz.

### 2.6. Analysis

To carry out the following reported analyses of this study, we used Microsoft Excel 2019 (Microsoft Corporation, 2019) and the statistical software R version 3.5.3 (R Core Team, 2019). The logistic regressions were performed using the R package “quickpsy” (Linares and López i Moliner, 2016). To fit the HLMs the R package “lme4” was used (Bates et al., 2015). To fit the bayesian HLMs the R package “brms” was used (Bürkner, 2016). The brms package implements bayesian HLMs in R using the probabilistic programming language “Stan” (Carpenter et al., 2017) under the hood, with an lme4-like syntax. Sorensen and Vasishth (2015) provide a detailed and accessible introduction to bayesian HLMs applied to cognitive science using Stan. The “Loo” package (Vehtari et al., 2017) was used to compare the bayesian models.

**Figure 3:**
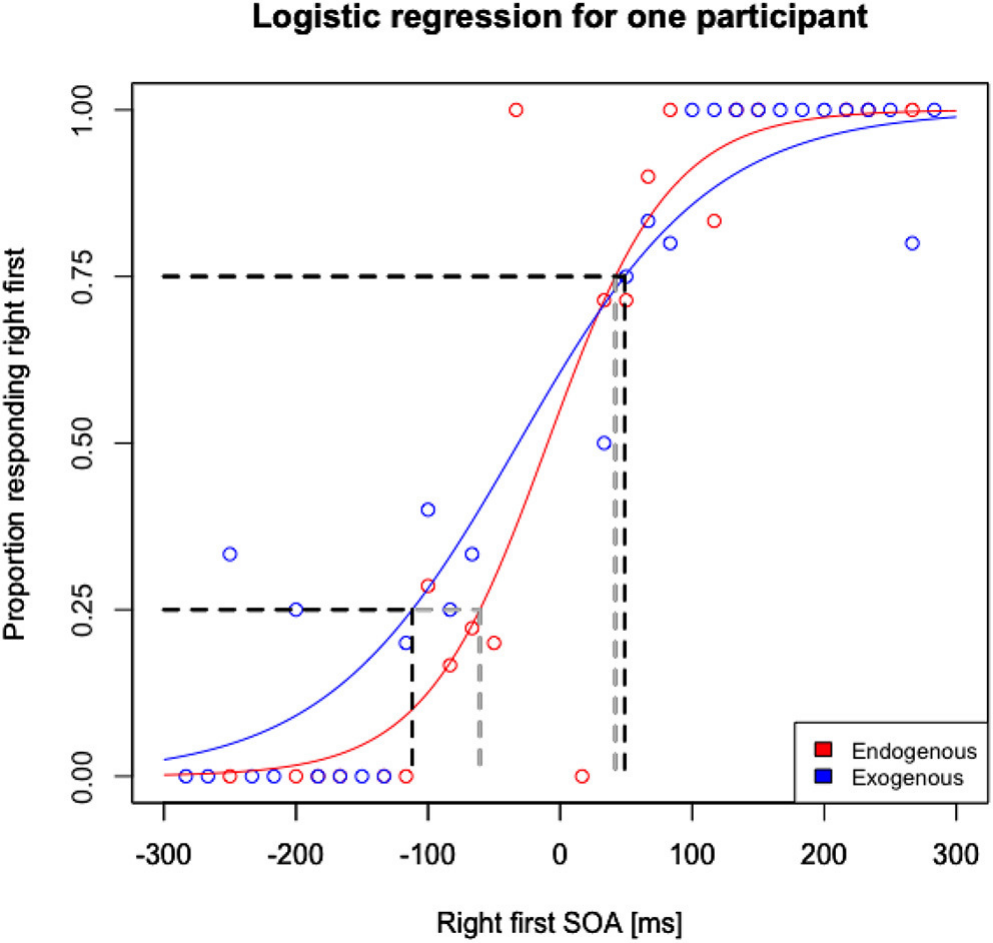
Proportion responding right first as a logistic regression of stimulus onset asynchrony (SOA) for one participant as an example. JND measurements were taken for the endogenous (red) and exogenous (blue) cued items by halving the distance between the SOAs corresponding to 0.75 and 0.25 proportions (see gray segments for endogenous cued items and black segments for exogenous cued items).

**Figure 4:**
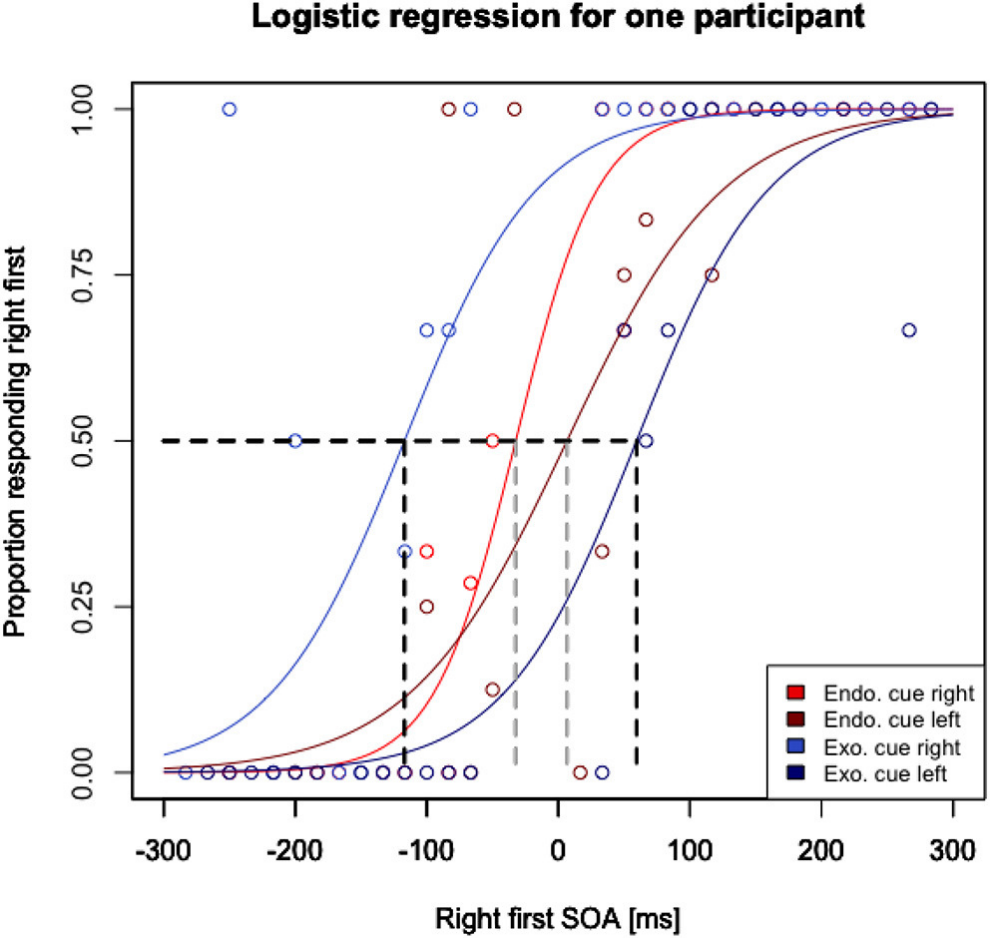
Proportion responding right first as a logistic regression of stimulus onset asynchrony (SOA) for one participant as an example. PSS measurements were taken for the endogenous right cued items (red), endogenous left cued items (dark red), exogenous right cued items (blue), and exogenous left cued items (dark blue) for the SOAs corresponding to 0.50 proportion (see gray segments for endogenous cued items and black segments for exogenous cued items). Notable is the larger gap between right cued items and left cued items for exogenous trials compared to endogenous (reflecting larger distraction by peripheral cues).

To estimate the JNDs and PSSs for each participant, we used a similar procedure as Lim and Sinnett (2011). At first, data from each participant was separated into endogenous cued trials, endogenous left or right cued trials, exogenous cued trials, and exogenous left or right cued trials. A logistic regression model was then fitted to each cue type for each participant's run, resulting in 36 models per participant, namely 12 JND scores (3 endo pre, 3 exo pre, 3 endo post and 3 exo post) and 24 PSS scores (3 endo pre right, 3 endo pre left, 3 exo pre right, 3 exo pre left, 3 endo post right, 3 endo post left, 3 exo post right and 3 exo post left). Two measures were then calculated for each participant's run. First, the JNDs were calculated independently for the endogenously and exogenously cued trials by taking the SOAs corresponding to 0.75 and 0.25 proportions, and then halving the distance between these SOAs (Figure 3). This halved distance is the specific JND for each condition (Endogenous/Exogenous) within participants for testing (pre/post), and session (1, 2, 3). Second, the PSSs were taken from the models of the right and left cued endogenous and exogenous trials for the SOAs corresponding to 0.50 proportion for right first responses (Figure 4). This is the point of maximal uncertainty, representing chance performance, and statistically, the PSS. To compare the PSS scores between the pre- and post-tasks, the distances between the left and right PSS separate for endogenous and exogenous pre and post measurements were calculated.

All estimated JNDs outside a range of 2.5 of the absolute deviations around the median (Leys et al., 2013) were excluded, due to response error rates and non-convergence of the algorithm fit, resulting in 39 data points being excluded (~12.4%). With regards to the PSS, the distances between the left and right cued PSSs of the pre task were compared to the distances between the left and right cued PSSs of the post task, separated by session. All responses outside a range of 2.5 absolute deviations around the median (Leys et al., 2013) of the PSS pre-post difference were excluded, due to response error rates and non-convergence of the algorithm fit, resulting in the exclusion of 37 data points (~11.8%).

In order to test the pre-registered hypothesis of whether flow has an influence on the JND, hierarchical linear models (HLMs) were calculated. These models estimate the influence of flow on the change (moderation) of the JND from pre- to post-testing. HLMs account for data hierarchies as observed in repeated measurement designs. In our study, repeated points of measurement are nested within sessions and within participants, and this data dependency can be recognized by HLMs.

In order to test whether the mean values of the nested groups differ and whether the differences justify a three-level model structure, a fully unconditional model was created. To test if there is a general difference in the JND from the pre- to post-testing a predictor was added to the model to estimate this relationship. Additionally, we included condition (endogenous vs. exogenous) as a predictor in the model to test the general contrast between these two conditions. Lastly, the interaction of the difference from pre- to post-testing and flow as a predictor was included in the model. A significant fixed interaction effect between flow and the pre- to post-testing TOJ would indicate a moderation of the change in the JND by flow from the pre- to the post-testing, and therefore a relationship between flow and the JND.

Stegmueller (2013) have shown that frequentistic approaches to modeling HLMs are sometimes susceptible to relatively small sample sizes, whereas bayesian probabilistic models appear to be more robust. In particular, the bayesian probabilistic approach shows considerably better properties with regard to the estimation of confidence intervals. Therefore, due to our relatively small sample size, for the main analysis of the dependent variable JND, an additional probabilistic model, with a bayesian point of view, was estimated to support the results. An interaction term of pre- to post-testing and flow which does not include zero in its 95% credible interval would indicate an effect of flow on the JND.

#### 2.6.1. Exploratory Analyses

In order to investigate whether the PSSs are influenced by the amount of experienced flow, HLMs for the dependent variable PSS were estimated in the same way as for the dependent variable JND. In order to test whether the JNDs and PSSs within the musicians differ from those of the athletes, a predictor that estimates this distinction was calculated. To test whether the relationship between the JND or PSS and flow is dependent on the response of the control question, an additional interaction term that estimates this relationship was built into the models.

## 3. Results

Across all three sessions, the mean of the global flow score as reported on the AFSS was 3.43 (out of 5), ranging between 1.58 and 4.85, suggesting that our participants did experience a broad variance of flow levels. The Cronbach's alpha coefficient for the AFSS was, α = 0.95. The mean values, standard deviations and Cronbach's alpha for the nine subscales can be found in Table 1. The mean answer on the 5-point Likert scale to the control question [i.e., “My state (body feelings, emotions and thoughts) during the task on the computer was similar to the state I had during my music/sports performance.”] was 3.33 (*SD* = 1.09), suggesting that participants tended to affirm the statement.

**Table 1:** Number of items, mean value, standard deviation, and Cronbach's α for the nine subscales of the AFSS.

**Subscale**	**No. items**	***M***	***S.D*.**	**Cronbach's α**
MAA	3	3.19	0.96	0.67
CG	3	3.68	1.14	0.91
CO	4	3.41	1.04	0.83
UF	2	3.47	1.06	0.77
CS	3	3.57	0.87	0.69
TT	3	3.24	0.96	0.68
CN	2	3.46	1.21	0.86
SC	3	3.49	1.14	0.83
AE	3	3.42	0.97	0.80

*n = 78; MAA, merging actions and awareness; CG, clear goals; CO, concentration on task at hand; UF, unambiguous feedback; CS, challenge skill balance; TT, transformation of time; CN, sense of control; SC, Loss of self-consciousness; AE, autotelic experience*.

### 3.1. Analysis of the JND

A model that estimates the explained variance by the nested groups showed that the subject effect (level 3) was responsible for 15% of the explained variance, and 38% of the JNDs variance was explained by session effects (level 2). This suggests the necessity of using a three-level model structure (pre-/post-testing nested in sessions within subjects).

When including pre-/post-testing of the JND as a fixed coefficient in the model, representing the estimated general difference between the measured JNDs before the practice or rehearsal session and after the practice or rehearsal session, the model's fit was significantly improved compared with the fully unconditional model, *X*^2^(1) = 4.82, *p* = 0.03. When including the factor pre-/post-testing as a random coefficient in the model (M1.1), there was no significant improvement in the model fit, *X*^2^(4) = 6.72, *p* = 0.15.

Including the discrimination of the conditions exogenous and endogenous as an additional fixed coefficient to our model to test for possible differences between these two conditions, the model's fit improved significantly, *X*^2^(1) = 19.66, *p* < 0.001. There was no significant improvement in the model fit, *X*^2^(3) = 7.73, *p* = 0.052, when including this predictor as a random coefficient.

To test whether flow is a moderator for the change of the JND between the pre- and the post-testing, an interaction term of the variable pre/post and flow was added as a fixed coefficient to the model (the coefficients of the model can be seen in Table 2). The interaction between the pre-/post-testing and flow amounts to −5.8 ms, *SE* = 3.02, 95% *CI* [−11.71 to 0.12], *t*_(206)_ = −1.92, *p* = 0.055. The model fit did not significantly improve as we added the interaction as a fixed coefficient, *X*^2^(2) = 3.64, *p* = 0.16.

**Table 2:** Estimated coefficients of the JND-model.

**Predictors**	**Estimate**	**Std. Error**	**CI**	***P***
(Intercept)	53.30	4.31	44.85 to 61.74	<0.001
Condition	10.42	2.26	−9.83 to −0.89	<0.001
Flow	2.84	3.44	−3.91 to 9.59	0.409
Prepost	−5.36	2.28	5.99 to 14.85	0.019
Prepost * Flow	-5.80	3.02	−11.71 to 0.12	0.055
**Random effects**				
Var: session:subject	152.93			
Var: subject	331.62			
σ^2^	343.18			
Num. obs.	274			
Num. groups: session: subject	77			
Num. groups: subject	27			

*CI, 95% Confidence Interval; Flow, Mean-centered Values*.

The estimated interaction of pre/post, flow and the condition (exogenous/endogenous) was not significant (*p* = 0.27), suggesting that there is no difference between the interaction of the pre-/post-testing and flow for the endogenous condition when compared with the exogenous task.

In regards to the JND, the interaction between pre/post and flow does not depend on the answer of the control question (*p* = 0.85). Thus, the connection between the values of the pre-/post-testing and flow is not stronger if the control question is answered higher.

#### 3.1.1. Probabilistic Modeling of the JND

**Figure 5:**
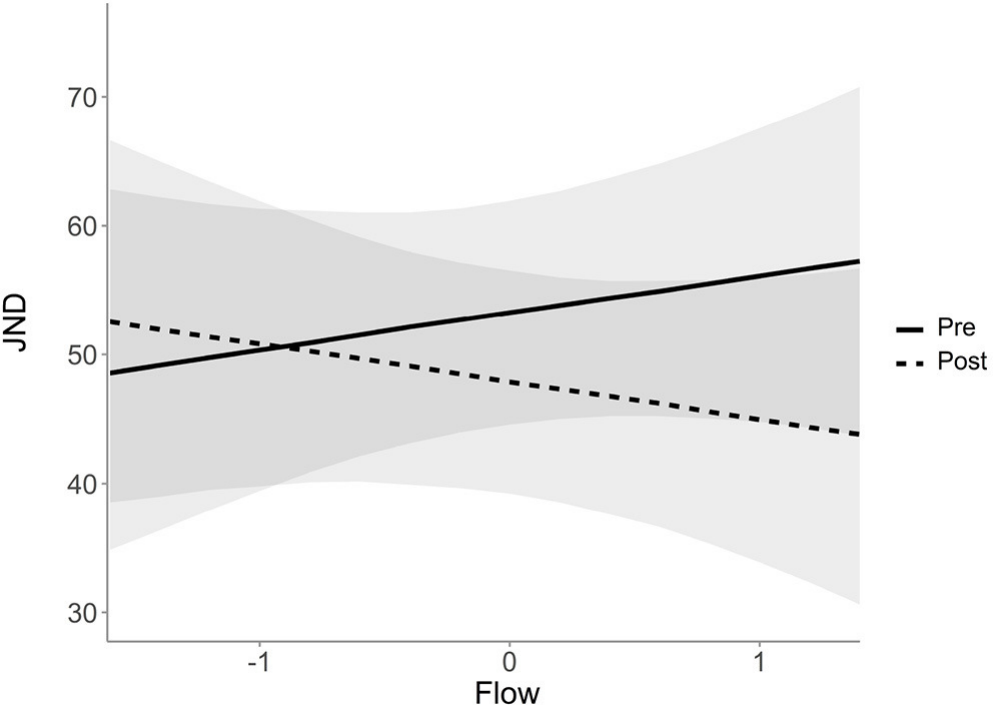
Estimated JND scores and their 95% credible intervals as a function of flow, divided into pre- and post-measured values.

The estimated interaction coefficient of Flow and the difference from pre- to post-testing within the bayesian model is −5.75 ms, *SE* = 3.04, 95% Credible Interval [−11.8 to −0.02]. The 95% credible interval can be interpreted in a way that there is a 0.95 probability that the value of the intercept lies between −11.82 and −0.02 ms. This effect is visualized in Figure 5. Low values of flow indicate a negative difference between the pre- and the post-testing and a high value of flow shows a positive difference between the pre- and the post-testing. It should be noted that the probabilistic estimate differs from the frequentistic estimate in terms of whether zero should be included in the confidence or the credible interval, respectively. The results of the estimates therefore do not seem to agree entirely on the extent to which the effect can be perceived as significant. Furthermore, a model which includes the interaction term of pre- to post-testing and flow differs only slightly from a model which does not include this predictor in terms of its predictive accuracy (elpd_diff = −0.2), and not larger than the standard error of these estimations (se_diff = 0.3). This indicates that a model which includes the interaction term of pre- to post-testing and flow does not make a substantial contribution to the prediction of new data.

### 3.2. Analysis of the PSS

A model that estimates the explained variance by the nested groups shows that 13.1% of the variance of the PSS was explained by participants. This indicates the necessity of a two-level-structure (pre-/post-testing nested within subjects). A three-level-structure (nesting the values of the PSS in sessions within participants) leads to an over-fitting of the estimated model, and therefore was not considered.

To test whether flow is a moderator for the change of the PSS between pre- and post-testing, an interaction term of the variable pre/post and flow was added to the model. As can be seen in Table 3, the estimation shows that a one unit change in the value of “flow” moderates the change from the pre- to the post-testing by −21.3 ms, *SE* = 7.28, 95% *CI* [−35.58 to −7.05], *t*_(227)_ = −2.93, *p* < 0.01. Essentially, this means that for every unit increase/decrease in experienced flow the PSS improved/diminished by 21 ms, respectively, indicating improved control of spatial attention with increased flow. This model also significantly accounted for the explained variance compared to a model without this interaction, *X*^2^(3) = 9.82, *p* = 0.02. Adding the effect of the interaction as a random effect leads to an over-fitting of the data.

**Table 3:** Estimated coefficients of the PSS-model.

**Predictors**	**Estimate**	**Std. Error**	**CI**	***P***
(Intercept)	41.12	5.36	30.61 to 51.62	<0.001
Condition	64.12	9.18	46.13 to 82.111	<0.001
Flow	12.21	5.79	0.86 to 23.57	0.035
Prepost	6.84	5.52	−3.98 to 17.65	0.215
Prepost * Flow	−21.31	7.28	−35.58 to −7.05	0.003
**Random Effects**				
Var: subject (Intercept)	153.05			
Var: subject Condition	1427.08			
Cov: subject (Intercept) Condition	373.90			
σ^2^	2053.72			
Num. obs.	276			
Num. groups: subject	27			

*CI, 95% Confidence Interval; Flow, Mean-centered Values*.

**Figure 6:**
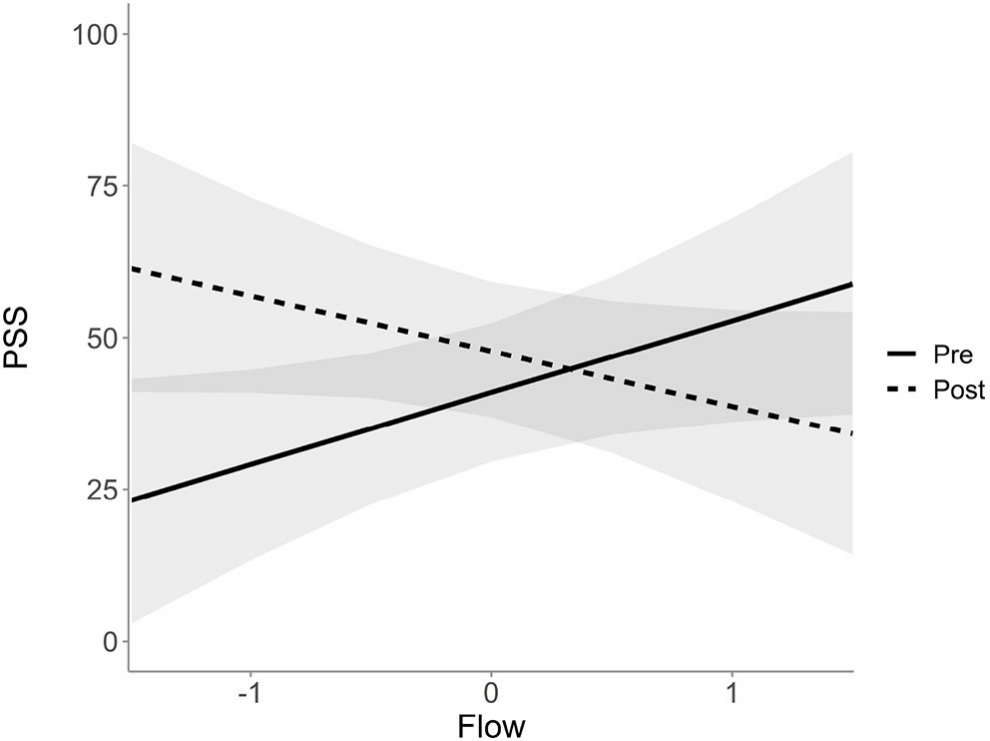
Estimated PSS scores and their 95% credible intervals as a function of flow, divided into pre- and post-measured values.

Probabilistic modeling of the data supports these results and estimates a coefficient of −20.9 ms, *SE* = 7.34, 95% *CI* [−35.06 to −6.41], for the interaction of flow and the change from pre- to post-testing. Figure 6 shows the relationship from pre to post PSS changes depending on flow. Lower flow values are associated with an increase in PSS values and high flow values are associated with a decrease in PSS values.

Including the interaction of pre/post, flow, and condition (exogenous/endogenous) to test if the moderation depends on the condition, there is neither an improvement of the explained variance [*X*^2^(3) = 1.32, *p* = 0.72] nor a significant three-way interaction [−13.93, *SE* = 14.50, 95% *CI* [−42.35 to 14.50], *t*_(226)_ = −0.96, *p* = 0.34]. This indicates that the moderation of the PSS by flow remains the same under both endogenous and exogenous conditions.

In regards to the PSS, the interaction between pre/post and flow does not depend on the answer of the control question (*p* = 0.98), suggesting that the connection between the values of the pre-/post-testing and flow is not stronger if the control question is answered higher.

### 3.3. Effects of the Domain on the JND and the PSS

When directly comparing the performance between musicians and athletes (Domain), there are no significant differences in the JND scores (*p* = 0.53). However, the rate of change from the pre- to the post-testing seems to differ significantly by 11.3 ms, *SE* = 4.82, 95% *CI* [1.89–20.79], *t*_(202)_ = 2.35, *p* = 0.020. Athletes have thus a 11.3 ms larger change from the pre- to the post-measurement compared to musicians. Regarding the PSS, no difference between musicians and athletes in general (*p* = 0.83) nor in their rate of change from pre-to post-testing (*p* = 0.57) was observed.

## 4. Discussion

Phenomenological differences in perception while performing a demanding task have been reported on several occasions. To the best of our knowledge, this is the first study to address the question of whether increased flow experience can improve perception, with the aim to investigate the relationship between modulated temporal and spatial visual processing at different time points and flow states in a within-subjects design. Of critical importance, the participants in this experiment participated over several experimental sessions, thereby enabling the measurement of a range of self-perceived flow states from which we can assess whether increased self-reported flow correlated with improved spatial and temporal processing, as measured by the TOJ task. This controls for the possibility that previous research addressing similar questions simply measured perceptual performance between more skilled and lesser skilled participants: Wesp et al. (2004) demonstrated that accomplished darts players perceived the target to be larger than novices with lower abilities, Witt and Proffitt (2005) suggested that softball players who were successful at hitting recalled the ball to be bigger than players with less success, Lee et al. (2012) claimed that people who perform better in archery see the target as larger than weaker archers, and Witt et al. (2008) show that golfers with high performance perceived the size of the cup to be bigger compared to lower performing golfers. Furthermore, possible short-term modulations in temporal perception like the speed of the ball in baseball (Gray, 2013) or in tennis (Witt and Sugovic, 2010) was perceived to be slower among better players. Nevertheless, these studies failed to take into account the intra-individual differences of the participants, therefore it is difficult to claim that flow states (i.e., when a participant is performing optimally) does lead to improved perception. In the present study, intra-individual differences in flow state were repeatedly measured, with our findings suggesting a flow state dependent correlation instead of a person dependent correlation.

The results of the present pre-registered study indicate a relationship between the value of experienced flow and spatiotemporal information processing. The improved performance was manifested in improved temporal perception (i.e., reduced JND scores) and significantly improved spatial attentional control (i.e., reduced PSS scores) when flow states were highest. In a recent longitudinal experiment by Cowley et al. (2019), where flow was induced with the help of a video game-like high-speed steering task, possible trial-wise fluctuations of performance due to flow were found, suggesting that performance was enhanced when participants experienced increased flow. These results dovetail with our findings, and might likewise indicate a short-term modulation of information processing and performance by flow.

### 4.1. Temporal Processing and Flow

The results indicate a positive correlation between the value of experienced flow and temporal processing. Specifically, when participants reported higher flow states in the practice or rehearsal session, they also performed better in the post-performance TOJ task compared with the pre-performance TOJ task, approximately by 5.8 ms per one-unit change of flow. But the results cannot draw a clear picture of the significance of the effect of flow on the JND. Although probabilistic models demonstrated that a coefficient of zero is not in their credible interval, the effect does not seem to contribute significantly in terms of predicting new data. In addition, the frequentistic model, although clearly pointing in one direction of supporting the alternative hypothesis, did not show a significant effect and the interaction term did not improve the fit of the model. Due to the way the data was collected (in the field), it can be assumed that a high level of noise in the data contributed to wide confidence intervals (and slightly smaller credible intervals), which makes it difficult to provide a reliable statement about the effect of flow on the JND and its strength.

On average, across all participants and independent from reported flow levels, the JND from pre- to post-testing generally improved (decreases) by approximately 5 ms. Despite there being no significant difference found overall when comparing the JND between athletes and musicians, there was a difference in the general rate of change from pre- to post-testing between the two domains. Specifically, athletes' JND scores deteriorated by 11 ms compared to musicians. This means that the musicians have improved JND scores while the athletes' performance has weakened. The differences between the rate of change of the two domains may be explained by the fact that athletes experience increased fatigue (compared to musicians) due to their physical performance while musicians might benefit more from learning effects and increased concentration, or, in contrast to the athletes, are not fatigued. However, these assumptions are purely speculative and the underpinning reason for these differences should be explored in further research. These differences were not observed in relation to the PSS.

### 4.2. Spatial Attention and Flow

In regards to spatial attention, it appears that increased flow experiences resulted in significant improvements in the PSS. More precisely, PSS improved by approximately 21 ms for each one unit increase in reported flow, suggesting that spatial attention was indeed modulated during flow experiences. It is important to note that this improvement could in fact be underestimated given that performance specific demands on spatial attention are arguably less for musicians than athletes (although, musicians are required to divide attention between their own performance and musical notes, and also the conductor and other fellow musicians). Future research could focus specifically on how flow experiences might affect attentional/perceptual mechanisms that are more tightly coupled with differing music/sport specific demands. Similar to Lim and Sinnett (2011) larger overall PSS scores (64ms difference) were observed for exogenous than for endogenous trials, which demonstrates that exogenous stimuli have a greater impact on attention. With direct respect to the perception of central objects (i.e., endogenous trials), it is likely that attentional focus is increased during flow experiences. This effect is reflected by participants' PSS scores, which indicate that a high level of flow reduces the distractibility from the central cues, resulting in a lower PSS.

### 4.3. Theoretical Implications

Attention plays a critical role in how time is experienced. Consider how a pleasant event (e.g., an excellent film or book) makes the subjective time seem to pass more quickly compared to something less pleasant (e.g., a boring lecture or perhaps this article for some). That is, the perception of time can be modulated depending on one's focus during the task (Phillips, 2012; Wearden, 2015). Arguably, the basis for time perception is rooted in the ability to process temporal order, with temporal resolution allowing for the successful recognition of stimulus order or simultaneity (Thönes and Stocker, 2019). Yet, the question remains as to how exactly the phenomenological perception of the passage of time during flow states and the processing of temporal information are related.

As a theoretical framework for the subjectively perceived slow down in time perception, Tse et al. (2004) might offer an alternative. These authors found that during unexpected events attention is arguably highly engaged, potentially leading to an increase in the amount of information processed during that time and to the subjectively experienced expansion of time. The authors conducted an oddball paradigm, in which participants were required to respond to a low-probability target that occurred within a range of high-probability stimuli. Participants were required to judge the duration of the presented low-probability target and decide whether it lasted longer or shorter than the standard items. The authors found that unexpected stimuli that were in fact presented for a shorter amount of time than the standard stimuli were, in fact, judged to be presented for the same length, suggesting that the engagement of attention for unexpected stimuli potentially leads to an increase in the amount of processed information, and subjectively perceived time. Essentially, more information is extracted from an unexpected signal. Arstila (2012) further posit why such faster rates of information processing lead to the experience of time as moving slower than usual. Specifically, these authors suggest that one component of the perception of time is determined by the speed of things in the external world. This re-afferent system plays a crucial role in determining the time that we perceive. The experience of faster acting than usual also implies that external objects might be slower than usual. Applying this logic to our study would suggest that participants who experienced a high flow state (in which attention plays a crucial role), processed more temporal information during the post TOJ task than in the pre task, and therefore, their JND scores improved (i.e., reduced). Moreover, we might realize that we are able to shift our attention from one stimulus to another more quickly than usual and therefore, our re-afferent system provides us with information that our internal processes are faster than normal.

One possible alternative explanation for our results could simply be that participants were subject to learning effects because of the repeated measures design. This learning effect could manifest itself within a session, between the pre- and the post-testing or across subsequent sessions. The change between the sessions is embedded in the hierarchical structure of the model, which accounts for the variance of this random effect. While a general modulation of JND does occur over sessions, these same differences were not observed for PSS. Moreover, it should be noted that the main interest of this study was not in the general effect of a single performance on the JND, which would be reflected in the change from pre- to post-testing, but whether this change is moderated by flow, for which the data suggest to be the case.

## 5. Limitations and Future Research

At this point it is impossible to make any statements regarding the direction of the causality of whether flow leads to improved perception, or if instead improved perception leads to flow. Thus, the question remains unanswered as to whether participants who experienced flow during the practice or rehearsal session have therefore an enhanced information processing, or if participants who show an enhanced information processing therefore experience higher flow states. While this concern should be partially alleviated given that our findings do imply that perception improves as flow increases *within* our participants, this is a question that should be elucidated by further research. Randomized controlled trials and experiments in which flow can be actively manipulated could provide insights into the causality relationships between flow states and spatiotemporal processing. Shehata et al. (2018) present a possibility to actively manipulate flow states and to induce them with the help of a "Music Rhythm Game," which arguably generates flow over the difficulty of the game (from “boredom” to “overload”). The difficulty in this is, of course, whether flow states caused by simple but controllable environments are similar or not to those experienced in self-chosen multifaceted sports and music environments. Another limitation that could be tackled with such a task, might be that the flow inducing sports or music performance was between the TOJ measurements and not at the same time.

An additional aspect of the current study that could be expanded in future research has to do with the inclusion of both athletes and musicians who play different sports and instruments, respectively. While this did result in differences between groups in terms of years of experience and training regimens, it should be noted that when including group as a factor no major differences were observed in the findings. We included different activities (i.e., athletes and musicians) in an attempt to more broadly explore how flow experiences from varying performance types might affect perception. Future research could consider exploring specific activities (e.g., only tennis players or only pianists) in order to see if perception is differently modulated based on the flow that is experienced from different activities.

Despite the inclusion of a control question to determine whether the state was approximately the same during the practice/rehearsal session and post task, and although the participants tended to agree with the control question, it is difficult to conclude that the flow-state experience was truly transferred to the post TOJ task. It was expected that there would be a stronger correlation between flow and pre- and post-measurement performance when the control question was answered higher. However, this was not the case. This could possibly be explained by the fact that the control question might not adequately assess whether the flow state of the participant extended to the TOJ task. In addition, although we attempted to keep the amount of time between music/sport session and testing as short as possible, memory biases could have influenced our results in that we presented the control question and the flow questionnaire at the end of the experimental trial and the items were answered retrospectively. In future investigations it would certainly be interesting to develop a task where flow and perception can be simultaneously measured, so as to completely avoid any potential memory confound. This could potentially be accomplished by using physiological measures, such as skin conductance, pulse rate (Tozman et al., 2015), brain activity or oculomotor indicators (Shehata et al., 2018). Knierim et al. (2018) provide an overview of peripheral nervous system indicators of flow. Furthermore, aspects that, in addition to the flow state, could also lead to an improvement in spatiotemporal processing, such as increased alertness after enhanced performance, should be controlled in further studies.

Due to the testing that took place in the field, the data are subject to high noise exposure, therefore the calculated models have difficulties in estimating accurate confidence/credible intervals. This limitation is notable as this potentially impedes an accurate determination of the effect size. For instance, and with particular regard to the pre-registered hypothesis about the moderation of the JND, no statistically significant and unambiguous answer could be found.

Lastly, even if the results regarding the modeling of the PSS may appear promising with regard to the understanding of the possibility that perception is modulated during flow states, the results were obtained in form of an exploratory analysis and should be confirmed in the sense of a pre-registered confirmatory analysis.

## 6. Conclusion

To the best of our knowledge the present study is the first to provide evidence that subjectively experienced improvements in perception during flow states are related to improved temporal and spatial visual processing. In this study, self-reported flow states and perceptual processing, as measured by a TOJ task, were obtained over several time points. The correlation between flow and the JND scores indicate an improved temporal processing during flow, with the correlation between flow and the PSS scores indicating significantly enhanced spatial attention. Combined, these findings provide evidence to suggest that anecdotal accounts of improved perception during flow states might actually reflect objective reality.

## Data Availability Statement

This study was pre-registered, in which the general procedure, the hypothesis for the confirmatory analysis about the relationship between flow and the JND, and the exploratory analysis about the relationship between flow and the PSS were documented. The pre-registration, “Transparent Changes” document, all generated data sets, experimental code, analyses and additional analyses are publicly available via the Open Science Framework: https://osf.io/xfrmu/.

## Ethics Statement

The studies involving human participants were reviewed and approved by Committee on Human Subjects, University of Hawaii at Manoa. The patients/participants provided their written informed consent to participate in this study.

## Author Contributions

SS and RA conceived the study. All authors participated in experimental design and decisions on the experiment specifications. RA, JJ, and SMS collected the data. JJ and SMS programmed the software for the task, recruited the participants, organized the open practice process, conducted the experiment, selected and applied the statistical analyses, interpreted the results, and drafted the paper. All authors participated in reviewing and revising the manuscript and approved the final version.

### Conflict of Interest

The authors declare that the research was conducted in the absence of any commercial or financial relationships that could be construed as a potential conflict of interest.
